# Functional Analysis of Familial Asp67Glu and Thr1051Ser *BRCA1* Mutations in Breast/Ovarian Carcinogenesis

**DOI:** 10.3390/ijms10094187

**Published:** 2009-09-24

**Authors:** Malinee Pongsavee, Pimpicha Patmasiriwat, Grady F. Saunders

**Affiliations:** 1 Department of Medical Technology, Faculty of Allied Health Sciences, Thammasat University, Rangsit Campus, Pathumthani, Thailand; 2 Department of Clinical Microscopy, Faculty of Medical Technology, Mahidol University, Salaya Campus, Nakornpathom, Thailand; E-Mail:mtppm@mahidol.ac.th (P.P.); 3 Department of Biochemistry and Molecular Biology, The University of Texas M.D. Anderson Cancer Center, Houston, TX 77030, USA

**Keywords:** *BRCA1* missense mutation, estrogen, estrogen receptor signaling pathway, breast/ovarian carcinogenesis

## Abstract

Estrogen is believed to be pre-initiator in the risk of breast cancer. The *BRCA1* is a tumor suppressor gene associated with breast and ovarian cancer risk. This report describes functional analysis of two *BRCA1* missense mutations (Asp67Glu and Thr1051Ser) observed in the familial breast/ovarian cancer patients in Thailand. Levels of luciferase activity of the two mutations were relatively lower than in the wild-type *BRCA1*. It is indicated that mutants may fail to promote the estrogen receptor dependent functions. It is presumed that estrogen and insulin/IGF-1 regulate c-Myc and cyclin D1 during breast cancer cell proliferation. It is also likely to affect ubiquitination mechanism. Since three affected cancer families carry the Asp67Glu mutation, it is believed that this type of mutation could have some effect on breast/ovarian cancer progression.

## 1. Introduction

Breast cancer is the most common form cancer of women worldwide. In many developing countries, including in Asia, which were formerly believed to have low risk of breast cancer, the incidence is presently increasing. *BRCA1* and *BRCA2* are the two tumor suppressor genes associated with breast and ovarian cancer risk [1]. *BRCA1* was localized on chromosome 17q21. The gene spans approximately 100 kb of genome, containing 22 coding and 2 noncoding exons. Its transcriptional 7.8 kb mRNA product encodes a 200 kD nuclear phosphoprotein consisting of 1,863 amino acids [2]. The *N*-terminus of BRCA1 contains a ring finger motif which is a cysteine and histidine rich (C3HC4) and domain capable of zinc binding. This zinc–binding ring finger domain is located close to the amino-terminus (residues 20–68) [2] and has ubiquitin ligase activity [3–4]. The *C*-terminal region of BRCA1 contains two carboxyl-terminal domains (BRCT domains). The BRCT domain is a phosphoprotein binding domain. BRCT domains interact with multiple transcription activators and co-repressors. The BRCT domain can interact with p53 and stimulates p53-dependent transcription of the p21 promoter. BRCA1 *C*-terminus interacts with RNA polymerase III holoenzyme in order to function as a transcriptional activator [5]. BRCT domain also has a role in the response to DNA damage [6]. The biological functions of *BRCA1* are the maintenance of genome stability and DNA repair. The other two functions are transcriptional activity and growth and differentiation [3–6]. BRCA1 is expressed in a number of tissues including breast and ovary and abundant in testis and thymus.

Estrogen plays important roles in the growth and differentiation of female secondary sex characteristics, in reproduction and in cellular metabolism [7,8]. The hormone may serve as the preinitiator and can alter the morphology of the mammary gland [9]. Once breast cancer initiation takes place, estrogen might promote the growth of transformed cells and lead to the development of breast cancer [10]. In addition, estrogen also increases proliferation and genetic instability by inducing free radical-mediated DNA damage and mutation [11].

Estrogens mediate their activity by interaction and activation of estrogen receptors (ER) [8]. Two forms of estrogen receptor, ER α and ER β are members of a nuclear receptor superfamily which responses to a variety of hydrophobic ligands such as steroid hormones (estrogens, glucocorticoids etc.), retinoic acid (vitamin A), vitamin D etc. [8]. Human ER α comprises of 595 amino acids and has molecular weight of 66–70 kD. It contains six functional domains (A to F). The A/B region contains activation function 1 (AF-1) which is important for transactivation and gene specificity. Region C is DNA binding domain (DBD) and contains two zinc finger motif and responsible for the binding of the receptor to estrogen response elements as well as contribute to dimerization and transactivation. Region D is the hinge region and contain nuclear localization. Region E contains the ligand binding domain (LBD) and activation function 2 (AF-2). AF-2 involves in transactivation in cooperation with AF-1. Human estrogen receptor β has 477 amino acids and is expressed in many tissues, including immune system and urogenital tract [12].

Upon hormone binding, the receptor undergoes physiochemical changes including phosphorylation at specific serine and tyrosine residues that are accompanied by conformational changes [12]. The transformed ER dimer binds to its specific estrogen response element (ERE) located in the promoter region of estrogen responsive genes, regulating their transcriptional activity. ER-ERE interactions are augmented by the binding of co-activators or co-repressors that further regulate gene transcription [13]. BRCA1 is a protein known to interact with and regulate activity of estrogen receptor α [14] as well as androgen receptor [15].

In Thailand, mutations in the *BRCA1* and *BRCA2* exons were found in cases of Thai familial breast/ovarian cancer, or isolated early-onset cancer. Asp67Glu and Thr1051Ser are the two *BRCA1* missense mutations which occurred in Thai familial breast/ovarian cancer patients [16]. The significance of these two mutations are unknown. Estrogen receptor signaling pathway is one of the pathways involved in breast/ovarian carcinogenesis. We studied the role of these two missense mutations in estrogen receptor signaling pathway for promote the breast/ovarian carcinogenesis.

## 2. Results and Discussion

**Figure 1.**
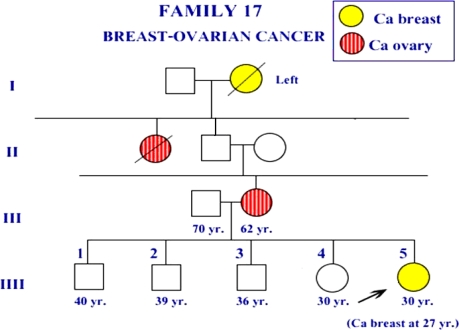
Pedigree of the breast cancer patient in F17. The patient had Asp67Glu *BRCA1* missense mutation.

**Figure 2.**
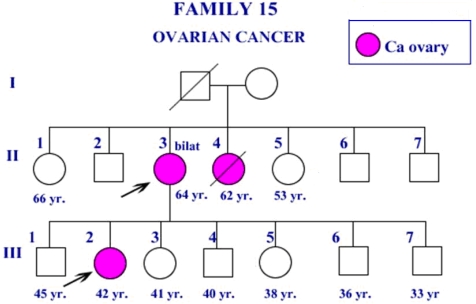
Pedigree of the ovarian cancer patient in F15. The patient had *BRCA1* Thr1051Ser missense mutation in addition to 3300delA frameshift mutation. Estrogen receptor and progesterone receptor were positive in this patient.

The report of Patmasiriwat *et al.* [16] showed that Asp67Glu and Thr1051Ser *BRCA1* missense mutations appeared in some of familial breast/ovarian cancer. Figure 1 and 2 showed the pedigree of the familial breast/ovarian cancer families which were found these two missense mutations. This report also showed that the patient in family F17 (ID34), the patient in family F18 (ID35) and one isolated early-onset breast cancer case (ID36) had Asp67Glu *BRCA1* mutation at exon 5. For the patient ID34 in family F17, the age of onset was low (27 years) and she had infiltrative adenocarcinoma of right breast. Both breast and ovarian cancer were found among the F17 members (Figure 1). Only breast cancer was found among the two members in family F18. Age of onset for the isolated breast cancer case (ID36) was 25 years.

The patient ID27 in family F15 carried *BRCA1* 3300delA-ter1061 at exon 11 and also had Thr1051Ser [16]. The age of onset was 42 years and she had bilateral ovarian serous papillary carcinoma. The clinical pathology showed that the estrogen receptor and progesterone receptor were positive in this case. The three members in F15 were ovarian cancer patients (Figure 2).

**Figure 3.**
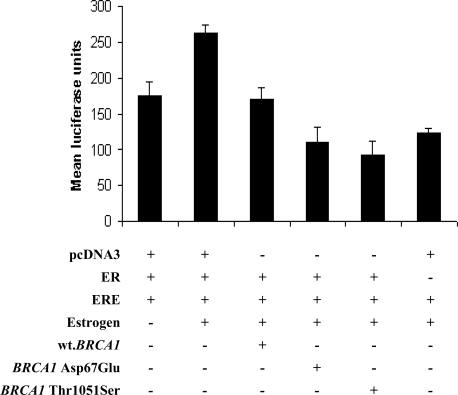
The role of *BRCA1* missense mutation, Asp67Glu and Thr1051Serine, to estrogen receptor signaling pathway. Wt.*BRCA1* expression vector and mutant *BRCA1* expression vectors were transfected into DU145 cell line for 48 hrs. Cells were stimulated by estrogen and assay for pERE-Elb-Luc reporter activity. The luciferase activity of *BRCA1* mutants were different from activity of the wild-type (*P* < 0.05). These missense mutations failed to promote the estrogen receptor signaling pathway for breast/ovarian carcinogenesis.

The role of Asp67Glu and Thr1051Ser on estrogen receptor signaling pathway were studied by transient transfection in DU145 cell line (prostate cancer cell line) [14] and luciferase activity was analysed. The first, second and sixth well were the proper control wells. Comparing luciferease activity of the six wells in this experiment, it showed that activity of the second well was the highest. The construct vectors of the second well include ER vector, ERE vector and estrogen, all components are necessary for estrogen receptor signaling. The first well had no estrogen and the sixth well had no ER vector. The luciferase activity of the first well was higher than the sixth well. From Figure 3, the average luciferase activity of the mutants (the fourth well and the fifth well) was significantly different from the wild-type *BRCA1 (*the third well) (*P* < 0.05). Luciferase activity levels of the two *BRCA1* missense mutations were relatively lower than the wild-type *BRCA1* (the third well). Estrogen receptor signaling pathway was inhibited in the presence of wild-type *BRCA1* in DU145 transfected cells as in the two mutants. In the vector construction in which ERE element was ligated to luciferase gene, level of lucifersase activity as response to ER in the presence of both *BRCA1* and estrogen was lower than the activity in system without *BRCA1*. Thus, our work showed that the two *BRCA1* missense mutations failed to promote the estrogen receptor signaling pathway in breast/ovarian carcinogenesis.

In Thailand, the missense mutations Asp67Glu and Thr1051Ser of *BRCA1* were previously detected in familial breast/ovarian cancer patients or isolated early onset cases. Asp67Glu missense mutation was occurred in three Thai breast/ovarian cancer patients. 3300delA-ter1061 frameshift mutation occurred in the other three Thai breast/ovarian cancer patients and two out of three patients had both Thr1051Ser missense mutation and 3300delA-ter1061 frameshift mutation occurred in the same person [16].

Estrogen is important for the development of female reproductive tissues, particularly the uterus and mammary glands [17] and plays a central role in the development and progression of human breast cancer [18]. The mechanism which estrogen stimulate cell proliferation is controversial. Couse *et al*. showed that it could activate estrogen receptor (ER) transcriptional activity while other investigators reported the non-transcriptional mechanisms by activation of intracellular signaling such as the pathway involving MAPK [19–20]. Estrogen induces mitogenic effects in breast epithelial cells by stimulating G_0_/G_1_ resting cells to enter the cell cycle and progress through the G_1_-S transition to complete a round of cell division [21–22].

Estrogen receptor (ER) is a candidate locus for familial late onset breast cancer susceptibility [14]. The high expression of ER in breast cancer is associated with responsiveness to hormonal treatment and a favorable prognosis. Mutation in the *ER* may modify the hormonal response in breast epithelium and potentially result in inherited susceptibility to breast cancer [23].

Estrogen binds and activates cytoplasmic ER α, which translocates to the nucleus, dimerizes, binds to estrogen responsive enhancer elements (EREs) on the target genes and activates transcription. Wild-type BRCA1 protein suppress estrogen-dependent mammary epithelial proliferation by inhibiting ER α–mediated transcriptional pathways related to cell proliferation [14]. The inhibition of estrogenic stimulation for mammary epithelial cell growth by BRCA1 contributes to breast cancer suppression. *BRCA1* function involves in estrogen receptor signaling pathway. Our study is in agreement with Fan *et al.* who showed that the wild-type BRCA1 inhibits estrogen receptor signaling pathway and may suppress estrogen-dependent mammary epithelial proliferation [14]. The loss of this inhibitory activity is believed to promote the proliferation of genetically damaged mammary epithelial cells. As proposed by Fan *et al.* and Zheng *et al.* [14,24], an inactivation of the ability to repress ER α transcriptional activity by *BRCA1* mutation may contribute mammary carcinogenesis.

Cyclin D1 is another factor which has been reported to involve in estrogen receptor function. Cyclin D1 protein is overexpressed in 25–80% of invasive ductal carcinoma cases. The protein is associated with poor prognosis in estrogen receptor α-positive cases [25]. Cyclin D1 is a downstream mediator of estrogen in MCF-7 cells [26,27]. Cyclin D1 also represents one of the key cell cycle regulators which is downstream of the PI3-kinase pathway and is required in addition to c-Myc to mediate the mitogenic effects of estrogen and insulin [28].

Studying about the role of two missense mutations in *BRCA1* (Asp67Glu, Thr1051Ser) to estrogen receptor signaling was investigated. Result showed that estrogen receptor signaling pathway was inhibited in the presence of *BRCA1* expression vector in DU145 cells. In the construct which ERE element ligated to luciferase gene, level of luciferase activity as response to ER in the presence of both estrogen and *BRCA1* was lower than the activity in system without *BRCA1*. Luciferase activity levels in the presence of the two expression vectors were also low, similar to the wild-type *BRCA1*. Thus the two *BRCA1* missense mutations could not promote the estrogen receptor signaling pathway for familial breast/ovarian carcinogenesis. These mutations may involve in familial breast/ovarian carcinogenesis in the other pathways. Breast/ovarian cancer might be occurred because of the other factors such as *c-Myc*, *cyclins,* insulin-like growth factor-I (IGF-I) etc. Estrogen and IGF-1 regulate c-Myc and cyclin D1 to stimulate breast cancer cell proliferation [28]. IGF-1 induces cyclin D1 and stabilizes the level of c-Myc and cyclin D1 through the PI3-kinase pathways. Estrogen activates the PI3-kinase and MAPK pathways by stimulating the autocrine growth factors. Cyclin D1 activates CDK4/6 while c-Myc induces the activation of cyclin E/CDK2 to stimulate maximal G_1_-S phase cell cycle progression [26,28–30]. These two missense mutations might promote estrogen and insulin/IGF-1 functions which regulate c-Myc and cyclin D1 for stimulate breast cancer cell proliferation.

The other pathway which these two missense mutations may involve in carcinogenesis is ubiquitination. The ring domains of BRCA1 (residues 1-109) are associated with BARD1 [4] and BAP1 [3]. BAP1 (BRCA1-associated protein) is a member of the ubiquitin *C*-terminal hydrolase (UCH) family and can bind BRCA1. UCH degrades ubiquitin. BRCA1 is also a ring finger protein that harbors E3 ubiquitin ligase activity and it is a part of an ubiquitin ligase complex [31,32]. The ubiquitin-proteosome pathway involves in regulating the stability of ring finger-containing proteins [3]. *In vitro*, the BRCA1 ubiquitin ligase activity requires interaction with two proteins. The *N*-terminus of BRCA1 interacts with BARD1 to form a heterodimer. The heterodimer has substantial E3 ubiquitin ligase activity with the UbcH5a class of E2 ubiquitin conjugating enzymes [33,34]. Loss of the activity occurs in some forms of BRCA1-mediated cancer, as disease-associated mutations that substitute Zn^2+^-binding residues within the BRCA1 ring affects BRCA1 ubiquitin ligase activity. This zinc–binding ring finger domain is located close to the amino-terminus (residues 20–68) [2] and Asp67Glu missense mutation is occurred in the amino-terminus. The missense mutation that predisposed to breast cancer cluster in the Zn^2+^- binding residues of the BRCA1 ring finger domain is critical to the ubiquitin ligase function [35–37] and the BRCA1 ubiquitin ligase function is linked to the DNA damage repair role of BRCA1 [38].

## 3. Experimental Section

### 3.1. Construction of vectors

The *BRCA1* Asp67Glu and Thr1051Ser expression vectors were created from wild-type *BRCA1* expression vector by using site directed mutagenesis kit (Strategene, Wilmington, DE, USA). The mutagenic primers for Asp67Glu *BRCA1* expression vector was forward: 5′-GT CCT TTA TGT AAG AAT GAG ATA ACC AAA AGG AGC C-3′ and reverse: 5′-G GCT CCT TTT GGT TAT CTC ATT CTT ACA TAA AGG AC-3′. The mutagenic primers for Thr1051Ser BRCA1 expression vector was forward: 5′-GAA GTA GGT TCC AGT AGT AAT GAA GTG GGC TCC AG-3′ and reverse: 5′-CT GGA GCC CAC TTC ATT ACT ACT GGA ACC TAC TTC-3′. The changed base in each missense mutation was underlined in the sequence of each forward primer detailed above. For site directed mutagenesis, 5 μL of 10 × reaction buffer, 10 ng of DNA template, 125 ng of forward primer, 125 ng of reverse primer, 1 μL of dNTP mix, 3 μL of QuickSolution, and ddH_2_O was added to a final volume of 50 μL. Then 1 μL of Pfu Turbo DNA polymerase (2.5 U/μL) was added. The PCR cycle was as followed: 1 cycle of 95 °C (1 min); 18 cycles of 95 °C (50 sec), 60 °C (50 sec) and 68 °C (1 min/kb of plasmid length); 1 cycle of 68 °C (7 mins). After the PCR cycling, placed the reaction tubes on ice for 2 mins. to cool the reaction for 37 °C or below. One μL of Dpn I restriction enzyme (10 U/μL.) was added and gently mixed the reaction mixture by up and down pipetting, then incubated at 37 °C for 1 hour. Two μL of the Dpn I-treated DNA was added in XL10-Gold ultracompetent cells, swirled the transformation reactions gently and incubated on ice for 30 mins. The reaction mixture was heat shocked at 42 °C for 30 sec and incubated on ice for 2 mins. The reaction mixture was added in LB media, incubated at 37 °C for 1 hour with shaking at 225–250 rpm. After incubation, 50 μL of the transformation mixture was plated on LB/ampicillin agar at 37 °C for 16 hours. The white colonies were picked up and cultured in LB/ampicillin media. They were incubated at 37 °C for 16 hours with shaking at 225–250 rpm, purified by Miniprep kit (Qiagen, Valencia, CA, USA) and sequenced by automated DNA sequencing.

### 3.2. Cell line and culture

DU145 (prostate cancer) cell line was used in this assay. DU145 was grown in DMEM/F-12 medium (Gibco BRL Life Technologies, San Diego, CA., USA) supplemented with 10% fetal calf serum and kept in a humidified 37 °C, 5% CO_2_ incubator.

### 3.3. Transient transfection

The vectors used in this assay were: (1) wild-type *BRCA1* expression vector, (2) Asp67Glu *BRCA1* expression vector, (3) Thr1051Ser *BRCA1* expression vector, (4) pERE-Elb-LUC vector as reporter gene, (4) RSV-ER expression vector, (5) pcDNA3 vector and (6) pSV40 β-galactosidase vector. DU145 cells at 50–70% of confluency in 24 well-cell culture plate were incubated for 24 hours. Six wells were used in this assay. Each well contained 10^5^ DU145 cells, and various combination of the vectors were added to each well as follows: the pERE-Elb-LUC vector, RSV-ER vector, pcDNA3 vector, pSV40 β-galactosidase vector in the first well; the pERE-Elb-LUC vector, RSV-ER vector, pcDNA3 vector, pSV40 β-galactosidase vector in the second well; the pERE-Elb-LUC vector, RSV-ER vector, wild-type *BRCA1* expression vector, pSV40 β-galactosidase vector in the third well; the pERE-Elb-LUC vector, RSV-ER vector, Asp67Glu *BRCA1* expression vector, pSV40 β-galactosidase vector in the fourth well; the pERE-Elb-LUC vector, RSV-ER vector, Thr1051Ser *BRCA1* expression vector, pSV40 β-galactosidase vector in the fifth well and the pERE-Elb-LUC vector, pcDNA3 vector, pSV40 β- galactosidase vector in the sixth well.

0.5 μg of each vector was used for transfection in serum free DMEM/F-12 containing Lipofectamine plus reagent (Invitrogen, Carlsbad, CA, USA) for 24 hours. After 24 hours, 1 μM estrogen (preparation by diluted with 2% charcoal stripped fetal bovine serum) was added in the well number second to the sixth, and incubated for 24 hours. The pSV40 β-galactosidase vector (Promega Corp., Madison, WI, USA) was added as an internal control. An equivalent amount of the pcDNA3 vector was used as the vector control. After estrogen was added and incubated for 24 hours, the transfected cells were washed twice with PBS. Luciferase and β-galactosidase assays were performed according to the manufacturer’s instruction. Luciferase activities were normalized in relative to β-galactosidase activity.

## 4. Conclusions

Our study concludes that the two *BRCA1* missense mutations failed to promote the estrogen receptor signaling pathway in familial breast/ovarian carcinogenesis. They may play as a mild cancer-risk modifier of some other defective mechanisms in cell cycle or ubiquitination and probably cooperate with these mechanisms to induce cancer in the breast/ovarian cancer patients.
